# Duality of Simplicity and Accuracy in QSPR: A Machine Learning Framework for Predicting Solubility of Selected Pharmaceutical Acids in Deep Eutectic Solvents

**DOI:** 10.3390/molecules30224361

**Published:** 2025-11-11

**Authors:** Piotr Cysewski, Tomasz Jeliński, Julia Giniewicz, Anna Kaźmierska, Maciej Przybyłek

**Affiliations:** Department of Physical Chemistry, Faculty of Pharmacy, Collegium Medicum in Bydgoszcz, Nicolaus Copernicus University in Toruń, Kurpińskiego 5, 85-950 Bydgoszcz, Poland; tomasz.jelinski@cm.umk.pl (T.J.); m.przybylek@cm.umk.pl (M.P.)

**Keywords:** solubility, deep eutectic solvents, machine learning, nuSVR, solubility prediction, COSMO-RS, molecular descriptors, mefenamic acid, niflumic acid

## Abstract

We present a systematic machine learning study of the solubility of diverse pharmaceutical acids in deep eutectic solvents (DESs). Using an automated Dual-Objective Optimization with Iterative feature pruning (DOO-IT) framework, we analyze a solubility dataset compiled from the literature for ten pharmaceutically important carboxylic acids and augment it with new measurements for mefenamic and niflumic acids in choline chloride- and menthol-based DESs, yielding N = 1020 data points. The data-driven multi-criterion measure is applied for final model selection among all collected accurate and parsimonious models. This three-step procedure enables extensive exploration of the model’s hyperspace and effective selection of models fulfilling notable accuracy, simplicity, and also persistency of the descriptors selected during model development. The dual-solution landscape clarifies the trade-off between complexity and cost in QSPR for DES systems and shows that physically meaningful energetic descriptors can replace or enhance explicit COSMO-RS predictions depending on the application.

## 1. Introduction

Carboxylic acids and their derivatives play an essential role in pharmacy as both active pharmaceutical ingredients and structural motifs in drug design [1,2,3]. Their carboxyl group enables proton dissociation, hydrogen bonding, and interactions with plasma proteins, which affect absorption, distribution, and overall bioavailability [4,5]. Among them, mefenamic and niflumic acids are representative nonsteroidal anti-inflammatory drugs (NSAIDs) [6,7], used clinically for pain and inflammation [8,9,10,11,12,13]; however, both exhibit limited aqueous solubility that complicates formulation and necessitates solubilization strategies [14,15,16]. Other acids included in this study, such as flufenamic [17,18,19], ibuprofen and ketoprofen [20,21,22,23,24], probenecid [25,26], and naturally occurring phenolic acids like ferulic, caffeic, p-coumaric, and syringic acids [5,27,28,29,30,31,32,33,34,35,36,37,38], span a broad chemical and functional space relevant to pharmaceutical and green solvent research.

Solubility remains a key physicochemical parameter influencing drug efficacy, processing, and environmental behavior [39,40]. Poorly soluble compounds require formulation innovations, increasing development costs [41,42]. Because solubility depends on multiple thermodynamic and molecular factors, including temperature, polymorphism, and solute–solvent interactions [43,44,45,46,47], predictive computational approaches are increasingly valuable. Among the various techniques used for the solubility enhancement of APIs [41,48,49], deep eutectic solvents (DESs) are particularly interesting and promising. DESs are a flexible and increasingly studied class of liquid systems formed by mixing appropriate hydrogen bond donors and acceptors, which leads to a significant reduction in the melting point relative to the starting components [50,51,52,53]. DESs are distinguished by a number of properties useful in a pharmaceutical context: low volatility (which promotes safety and reduces emissions), a wide spectrum of polarity and acidity, significant “tunability” through component selection, and the ability to solubilize compounds of various chemical natures [54,55]. In pharmacy, DESs are being researched as extraction media for isolating natural products, as potential carriers for formulations that enhance the solubility and bioavailability of APIs, and as so-called THEDES (therapeutic deep eutectic solvents), i.e., systems in which the components themselves may have biological activity or facilitate drug stabilization and delivery [56,57,58,59]. At the same time, the vast combinatorial space of possible components and molar ratios necessitates the use of accelerated testing methodologies and predictive tools, as an empirical review of all variants is costly and time-consuming.

In this context, computational solubility prediction, especially using QSPR and advanced machine learning (ML) methods, is becoming a strategic tool for both pharmaceutical development and green solvent design [60,61,62,63]. QSPR models establish mathematical relationships between molecular structure and macroscopic properties, providing interpretable links between descriptors and solubility behavior [64,65]. Modern ML algorithms, such as neural networks, graph-based approaches, support vector machines, and ensemble strategies, extend this paradigm by capturing complex, non-linear patterns and multidimensional interactions that are often inaccessible to traditional regression models [66,67,68,69]. Neural networks, in particular, can integrate heterogeneous representations including physicochemical descriptors, molecular fingerprints, graph embeddings, and solvent composition features, allowing them to learn transferable correlations across chemically diverse datasets [70,71,72]. Recent studies demonstrate that such ML-based approaches can successfully predict aqueous and non-aqueous solubilities of drug-like molecules, outperforming empirical correlations and physics-based models when trained on sufficiently broad and curated datasets [73,74,75]. Beyond accuracy, however, the practical value of predictive modeling lies in its ability to guide experiment planning, identify anomalous data, and support rational solvent selection. For this purpose, hybrid strategies that combine physically meaningful descriptors, derived from quantum-chemical or COSMO-RS computations, with data-driven optimization provide a balanced route toward robust, interpretable, and generalizable models. Such hybrid approaches are particularly relevant for complex solvent systems like deep eutectic solvents (DESs), where solute–solvent interactions are governed by multiple competing forces and explicit simulation is computationally demanding. The integration of COSMO-RS-derived descriptors with machine learning optimization therefore enables data-efficient exploration of the chemical space and identification of the key physicochemical drivers of solubility. At the same time, the issue of balancing model interpretability and predictive power remains central. Reliable solubility modeling requires rigorous feature selection, cross-validation, and uncertainty estimation to avoid overfitting and ensure transferability to new chemical systems.

The purpose of this work was to create a predictive model for estimating the solubility of various pharmaceutically active carboxylic acids. The model was developed by combining COSMO-RS-derived molecular descriptors with machine learning methods, based on our newly established DOO-IT (Dual-Objective Optimization with ITerative feature pruning) framework. New experimental data for mefenamic acid and niflumic acid were obtained for this study, which were supplemented with solubility values found in the literature for a number of acids used in the pharmaceutical realm. The constructed models were thoroughly validated, and their performance was discussed, highlighting their effectiveness and the potential for generalization.

## 2. Results and Discussion

### 2.1. Solubility Measurements of Mefenamic Acid and Niflumic Acid

**Figure 1:**
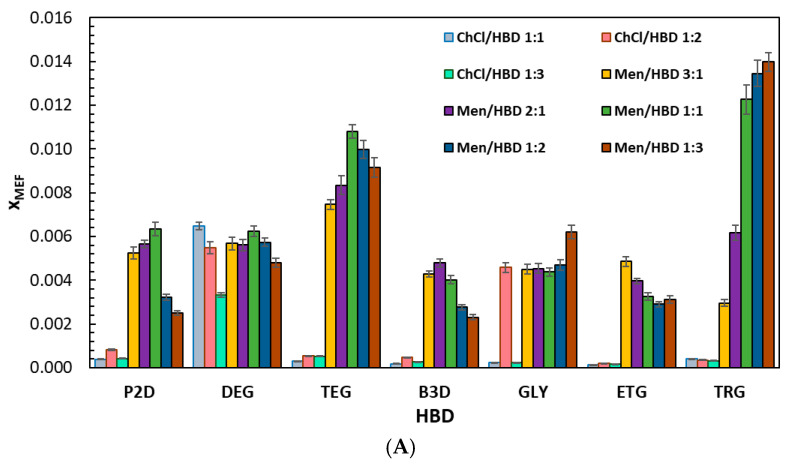

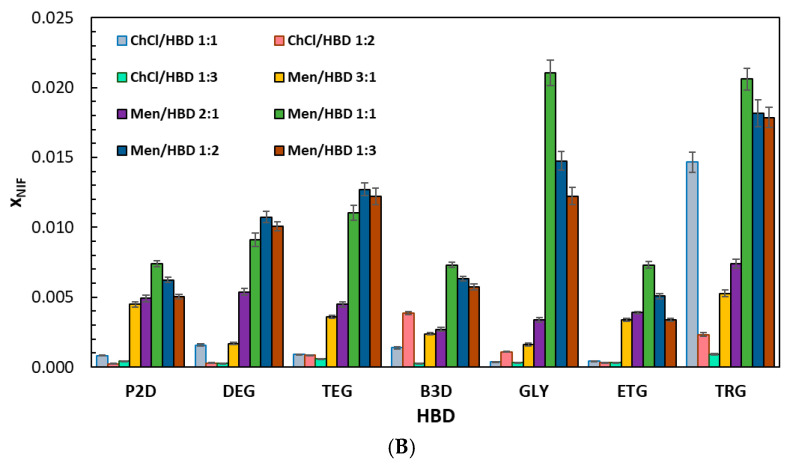
Experimental mole fraction solubility of (**A**) mefenamic acid and (**B**) niflumic acid in choline chloride- or menthol-containing deep eutectic solvents (DESs) measured at 25 °C.

Figure 1 summarizes the mole fraction solubilities of mefenamic acid (x_MEF_) and niflumic acid (x_NIF_) at 25 °C in choline chloride- and menthol-containing DESs, with full numerical values given in Table S1 (x_MEF_) and Table S2 (x_NIF_). Across all systems, x_MEF_ spans 1.38 × 10^−4^ to 1.40 × 10^−2^ and x_NIF_ 2.38 × 10^−4^ to 2.11 × 10^−2^, and menthol-based DESs generally afford higher solubility than their choline chloride analogs for both compounds. The highest x_MEF_ is observed for Men/TRG 1:3 (1.40 × 10^−2^), with Men/TEG also giving elevated values, while the highest x_NIF_ appears for Men/GLY 1:1 (2.11 × 10^−2^) and remains high in Men/TRG 1:1–1:3. Within the choline chloride series, ChCl/DEG 1:1 provides the top x_MEF_ (6.49 × 10^−3^), and ChCl/TRG 1:1 gives the top x_NIF_ (1.47 × 10^−2^). Considering the hydrogen bond donors (HBDs), TRG and TEG are associated with the largest solubilities in the menthol series, GLY is particularly favorable for x_NIF_ at 1:1, and DEG stands out for x_MEF_ in the choline chloride series; the effect of the HBD fraction is system-specific; for example, x_MEF_ increases from 3:1 to 1:3 in Men/TRG, x_NIF_ maximizes at 1:1 in Men/GLY, and values decrease beyond 1:1 in ChCl/DEG (x_MEF_) and ChCl/TRG (x_NIF_).

### 2.2. Identifying an Optimal Predictive Model via the DOO-IT Framework

**Figure 2:**
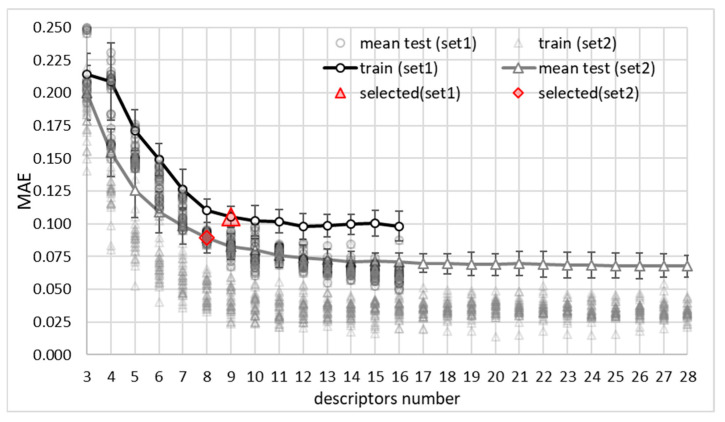
Stability analysis of the DOO-IT model selection workflow for predicting the solubility of pharmaceutical acids in DESs. The figure illustrates the variation in MEA (mean average error) values obtained for the pre-selected models based on Pareto fronts generated for every run covering the whole span of descriptors (16 for set 1 and 28 for set 2). Dots represent values obtained for train subsets and the solid line characterizes the mean test values with standard deviations. The points marked with a red triangle or diamond are the models passed by the multi-criteria selection framework. These models represent two alternative “basins of excellence” corresponding to either nine (set 1) or eight (set 2) descriptors balancing accuracy, parsimony, and stability.

The DOO-IT framework, aiming to find the most accurate and parsimonious machine learning model, was applied to the solubility dataset collected from our previous studies augmented with the new measurements of pharmaceutical acids in deep eutectic solvents provided in this work. In total, N = 1020 points characterized the solubility of ten pharmaceutical acids in choline chloride- and menthol-based deep eutectic solvents. The DOO-IT pipeline was performed 50 times, enabling the determination of statistically significant populations of dominating models. The results of the application of the entire three-pillar pipeline are provided in Figure 2. This is the central pillar of model development, visualizing the outcome of the DOO-IT model selection workflow.

The most significant finding illustrated in Figure 2 is that two different sets lead to distinct “basins of excellence,” highlighted by points marked in red color. This discovery directly refers to the “duality” in our article’s title, emphasizing that not a single “best” model is presented, but a strategic choice between two high-performing, yet fundamentally different, modeling approaches. Indeed, set 2 utilizes both energetic contributions and σ-potential distributions and this extended source of information results in a slightly simpler model (eight descriptors) in terms of numbers of descriptors (MAE_TEST_ = 0.0893 ± 0.0116, R^2^_TEST_ = 0.968 ± 0.052). On the other hand, utilization of set 1 exclusively for model development led to an only slightly worse model (MAE_TEST_ = 0.1054 ± 0.0082, R^2^_TEST_ = 0.944 ± 0.015) at the cost of an increase in the number of descriptors (nine). This outcome indicates that distinct, scientifically meaningful descriptor combinations can achieve competitive performance via different mechanisms. Two separate models can therefore be regarded as the ones with optimal performance, depending on the intended task. The nine-descriptor model captures the main drivers of solubility in terms of energetic contribution only. Augmenting the model with additional information relying on the charge density contributions to σ-potentials enables both the simplification of the model and making it more robust. This duality is an important finding suggesting a nuanced interplay between information used for model development and the resulting interchange of accuracy–parsimony–stability. The robustness and usefulness of the developed DOO-IT framework rely not only on the ability to generate a valuable model extracted from the vast universe of potential models but also to tailor it to a given dataset and descriptor combination using objective criteria and full automation, preventing biases.

**Figure 3:**
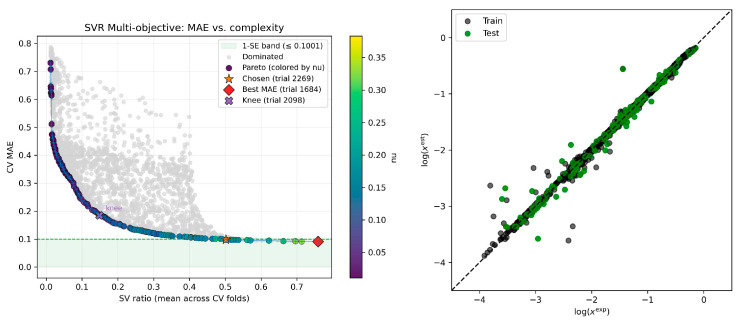
Dual-objective optimization and tentative model selection for the eight-descriptor model. The figure shows the balance between predictive accuracy (CV MAE) and model complexity (SV ratio). Each point corresponds to a distinct nuSVR model, while the Pareto front (dark purple points, shaded by the nu hyperparameter) marks the set of non-dominated solutions. The final model (trial 2269, orange star) was chosen according to the one standard error (1-SE) rule, which selects the least complex model falling within the 1-SE performance band (green region) of the best-performing candidate (trial 1684, red diamond). The parity plot in the right panel of the figure shows the agreement between the experimental (exp) and estimated (est) values of logarithmic mole fraction solubility (log(x)) for the selected optimal model. The following model parameters were found from the DOO-IT framework: {‘nu’: 0.25437165084768737, ‘C’: 33.95751326829903, and ‘log10_gamma_scale’: 0.4526771971941057}.

The first basin, located at eight descriptors, utilizes the following descriptors ordered in decreasing importance provided in brackets: E_vdW,API_ (0.69), ΔHBA1 (0.66), log(x_API_^COSMO^) (0.55), μ_API_ (0.51), ΔHH1, (0.46), ΔHH4 (0.43), E_HB,API_ (0.36), and ΔHH2 (0.32). These descriptors represent the most fundamental and universal properties of the organic acid molecules that dictate their behavior in DESs. Hence, for the studied set of data, the dispersion contribution of API and relative hydrophobicity values are the most dominant contributions to the solubility. Also, the relative value of acceptability at vicinal σ-potential regions is important. The model performance is provided in Figure 3. The plots highlight the inherent trade-off between accuracy and model complexity. The collection of non-dominated solutions forms a clear Pareto front (dark purple points), which represents the best accuracy attainable at any given level of complexity. Models lying to the right of this front (gray points) are inferior, since a simpler and more accurate alternative exists. Coloring of the points along the Pareto front according to the nu hyperparameter reveals a consistent pattern: lower nu values yield simpler models with reduced SV ratios, whereas higher nu values give rise to more complex models characterized by larger SV ratios.

**Figure 4:**
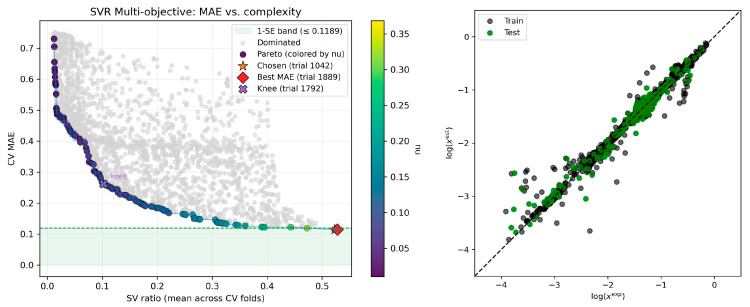
Dual-objective optimization and tentative model selection for the nine-descriptor model. The figure shows the balance between predictive accuracy (CV MAE) and model complexity (SV ratio). Each point corresponds to a distinct nuSVR model, while the Pareto front (dark purple points, shaded by the nu hyperparameter) marks the set of non-dominated solutions. The final model (trial 1042, orange star) was chosen according to the one standard error (1-SE) rule, which selects the least complex model falling within the 1-SE performance band (green region) of the best-performing candidate (trial 1889, red diamond). The parity plot in the right panel of the figure shows the agreement between the experimental (exp) and estimated (est) values of logarithmic mole fraction solubility (log(x)) for the selected optimal model. The following model parameters were found from the DOO-IT framework: {‘nu’: 0.257891586205508, ‘C’: 57.25987010428314, and ‘log10_gamma_scale’: 0.936163953105186}.

The second high-performance model utilizes the following nine descriptors, namely ΔE_Misfit_ (1.46), ΔE_vdW_ (1.15) log(x_API_^COSMO^) (0.94), E_vdW,API_ (0.72), E_HB,API_ (0.71), E_tot,API_ (0.66), μ_API_ (0.55), ΔE_tot_ (0.47), and E_Misfit,DES_ (0.47). This set of descriptors reveals the complex nature of solute–DES interactions under saturated conditions and the necessity of extending the core features of the former model with the additional polarity hydrogen bonding capacity of the solute and solvent. Hence, the extended nine-descriptor solution integrates a broader set of energetic and interaction terms to appropriately capture solvation phenomena. Detailed presentations along model hyperparameters are collected in Figure 4.

It is imperative to contextualize these findings within a crucial methodological framework. The dataset exclusively comprises organic acids of pharmaceutical relevance, including well-known compounds such as ketoprofen, ibuprofen, ferulic acid, probenecid, caffeic acid, p-coumaric acid, syringic acid, and flufenamic acid. A significant and unresolved challenge in modeling such systems is determining the dissociation state of acidic solutes. This is because their intrinsic acidity, quantified by pKa, is strongly modulated by the complex, non-aqueous environment of deep eutectic solvents (DESs). Lacking a reliable method to determine the precise ionization state of each acid in every unique DES, we adopted a necessary and consistent simplification: all molecular descriptors were calculated for the neutral, non-dissociated forms of the molecules. Despite this crude simplification, the remarkable accuracy of the resulting models is particularly noteworthy. It strongly suggests that the fundamental physicochemical properties of the parent molecule are the dominant drivers of solubility and that our modeling framework is robust enough to capture these governing relationships despite the simplification of the solute’s ionization state.

## 3. Materials and Methods

### 3.1. Materials

Mefenamic acid and niflumic acid (both ≥97%, Sigma-Aldrich, St. Louis, MO, USA) were used as received. The hydrogen bond acceptors were choline chloride (ChCl, CAS 67-48-1, ≥99%) and menthol (Men, CAS 89-78-1, ≥98.5%), and the hydrogen bond donors comprised ethylene glycol (ETG, CAS 107-21-1), diethylene glycol (DEG, CAS 111-46-6), triethylene glycol (TEG, CAS 112-27-6), tetraethylene glycol (TRG, CAS 112-60-7), glycerol (GLY, CAS 56-81-5), 1,2-propanediol (P2D, CAS 57-55-6), and 1,3-butanediol (B3D, CAS 107-88-0); all polyols/polyethers were obtained from Sigma-Aldrich with stated purity ≥ 99%. Methanol (analytical grade, CAS 67-56-1; Chempur, Piekary Śląskie, Poland) was used for sample handling where applicable. Unless otherwise specified, all chemicals were employed without additional purification.

### 3.2. Solubility Measurement Procedure

A similar methodology to [76] was employed, adapted here for mefenamic acid (MEF) and niflumic acid (NIF). Each DES was prepared at the target molar ratio by gentle heating with stirring until a clear single phase formed, then equilibrated to 25 °C. Pre-equilibrated aliquots were spiked with an excess of MEF or NIF, sealed, and agitated isothermally for 24 h at approximately 60 rpm. After equilibration, suspensions were maintained at 25 °C and supernatants were withdrawn, filtered through 0.22 μm PTFE syringe filters, and analyzed by UV–Vis. Spectra were collected from 200 to 500 nm in quartz cuvettes; analytical wavelengths were set at the absorption maxima (λ^MEF^_max_ = 351 nm; λ^NIF^_max_ = 344 nm). Concentrations obtained from UV–Vis were converted to mole fraction solubilities (x_MEF_ or x_NIF_) using molar masses and the density of each saturated solution; densities were determined gravimetrically at 25 °C. All measurements were performed in triplicate.

Calibration curves were established for each compound using methanolic stock solutions and serial dilutions. The characteristics for MEF were as follows: calibration range of 0.002 to 0.078 mg mL^−1^, slope of 28.265, intercept of −0.010, linearity of R^2^ = 0.9993, LOD = 0.00261 mg mL^−1^, and LOQ = 0.00790 mg mL^−1^. The characteristics for NIF were as follows: calibration range of 0.005 to 0.090 mg mL^−1^, slope of 18.808, intercept of −0.006, linearity of R^2^ = 0.9994, LOD = 0.00272 mg mL^−1^, and LOQ = 0.00825 mg mL^−1^.

### 3.3. COSMO-RS Computations

Application of the COSMO-RS framework [77,78,79,80,81] requires appropriate representation of molecular diversity. This is achieved by performing conformational analysis prior to the determination of any thermodynamic properties. For this purpose, the default protocol was applied, taking advantage of the COSMOconf (version 2023, BIOVIA COSMOlogic)/TURBOMOLE (version 7.7, 2023, TURBOMOLE GmbH) tandem for the generation of the most representative structures for all solutes and solvent molecules. The applied protocol is consistent with previously published schemes [82,83,84]. For each molecule, up to ten low-energy conformations were determined for both gas and condensed phases, the latter accounting for solvent effects under the conductor-like screening model. The resulting “cosmo” and “energy” files were generated using the BP_TZVPD_FINE_24.ctd parameter set, essential for thermodynamic calculations in COSMOtherm, which requires application of the RI-BP/TZVP//TZVPD-FINE level of theory.

### 3.4. Molecular Descriptors

Two distinct sets of molecular descriptors were generated using the COSMO-RS theory. The first set of descriptors comprised interaction energies from solubility calculations [82,83,84]. While the standard iterative solubility protocol is typically sufficient, it frequently yields erroneous predictions of complete miscibility for highly soluble solutes in DES systems [85,86,87,88]. For these cases, complete solid–liquid equilibrium (SLE) calculations were mandated. Requisite thermodynamic fusion data for the solid solutes, including melting temperature, T_m_, and enthalpy of fusion, ΔH_fus_, were obtained by averaging the available literature values [89]. The heat capacity of fusion was approximated as constant, ΔC_p,fus_ ≈ ΔS_fus_ ≈ ΔH_fus_/T_m_. The resulting Gibbs free energies of fusion values, ΔG_fus_ = ΔH_fus_−TΔS_fus_, utilized in the calculations are provided in the Supplementary Materials. The COSMO-RS output files yielded five primary descriptors for each solute: total intermolecular interaction energy, E_int,API_; its constituent electrostatic misfit, E_misfit,API_; hydrogen bonding, E_HB,API_; and van der Waals, E_vdW,API_, contributions, as well as the chemical potential and μ_API_. Analogous descriptors for the DES (E_int,DES_, E_misfit,DES_, E_HB,DES_, E_vdW,DES_, and μ_DES_) were calculated as the sum of the individual DES components, weighted by their respective molar fractions in the solute-free mixture. Relative descriptors, defined as the difference between the solute and DES values, were also included. Additionally, the computed solubility values from COSMO-RS were similarly included, log(x_API_^COSMO^).

Apart from this, the final set of molecular descriptors was augmented with values derived from σ-potential distributions. The standard COSMO-RS output consisted of 61 data points covering the charge density range of −0.03e/Å2 ÷ +0.03e/Å2. Consistent with prior machine learning applications, this data was reduced by averaging values over 0.005 intervals. This process resulted in a 12-step function defining three characteristic regions of the σ-potential: hydrogen bond donor (HBD1 ÷ 4, −0.03e/Å^2^ to −0.01 e/Å^2^), hydrophobicity (HH1 ÷ 4, from −0.01e/Å^2^ to +0.01 e/Å^2^), and acceptability (hydrogen bond acceptor, HBA1 ÷ 4, from +0.01e/Å^2^ to +0.03 e/Å^2^). Consequently, four descriptors were generated for each region, leading to twenty-four descriptors of this type for the solute, the solvent, and the relative difference between them.

### 3.5. Dataset

The values of molecular descriptors were computed for all components of studied systems, including pharmaceutical acids and DES constituents. The set of solutes included compounds for which new solubility measurements were included in this paper, namely mefenamic acid and niflumic acid. In addition, the values of the already published solubility data of ketoprofen, ibuprofen, ferulic acid, probenecid, caffeic acid, p-coumaric acid, syringic acid, and flufenamic acid in DESs were included [76,82,83,90]. In total, the dataset comprised N = 1020 mole fractions at saturated conditions in choline chloride-, betaine-, and menthol-based DESs with a variety of proportions of different HBA counterparts. Both dry and water-diluted systems were included if available. All data, including solubility values, fusion data, computed solubility, and all molecular descriptors, are available in the Supplementary Materials.

### 3.6. Machine Learning Protocol

#### 3.6.1. Core Algorithm and Data Preprocessing

The machine learning workflow was centered on the nu-support vector regression (nuSVR) algorithm [91], chosen for its demonstrated ability to effectively model complex, non-linear relationships often present in QSPR studies [92,93,94]. To handle these non-linearities, the Radial Basis Function (RBF) kernel was selected. The RBF kernel is a powerful and flexible choice, capable of mapping features into an infinite-dimensional space, which allows it to model intricate decision boundaries while requiring the tuning of only a single parameter: gamma. The optimization of the nuSVR hyperparameters was conducted as follows: The regularization parameter C and the nu parameter were directly optimized. The kernel coefficient gamma, which dictates the influence of each support vector, was optimized via a guided, data-driven strategy. For each optimization cycle, a baseline gamma_base value was heuristically determined from the median pairwise squared Euclidean distance of the training data subset [95,96]. The optimizer then refined this anchor by searching for an optimal logarithmic scaling factor. This approach focused the search on a physically relevant scale, enhancing optimization efficiency. Prior to model training, two standard preprocessing steps were performed. First, the full dataset (N = 1020) was partitioned into a training set (80%) and a held-out test set (20%) using a fixed random seed to ensure reproducibility. This was performed in non-deterministic fashion, enabling the exploration of different splits for every run using a random number as a seed for splitting. Second, all molecular descriptors in the training set were standardized by removing the mean and scaling to unit variance using the StandardScaler from scikit-learn [97,98]. As SVR algorithms are sensitive to feature scaling, this step ensured that no single descriptor disproportionately influenced the model due to its magnitude. The same scaling transformation was subsequently applied to the test set.

#### 3.6.2. Dual-Objective Optimization Protocol

To explicitly manage the inherent trade-off between model accuracy and simplicity, a dual-objective optimization (DOO) strategy was implemented using the Optuna framework (v. 3.2) [99,100,101]. The TPE sampler within Optuna was configured to simultaneously minimize two competing objectives, which were evaluated using a 5-fold cross-validation scheme on the training data. The first objective was predictive accuracy, quantified by the Mean Absolute Error (MAE). The second objective was model complexity, quantified by the mean support vector (SV) ratio. The SV ratio was calculated for each fold as the number of support vectors divided by the number of training samples in that fold, providing an intrinsic measure of complexity for nuSVR models. A model with a lower SV ratio was considered more parsimonious.

Hence, the outcome of dual-objective optimization is a set of solutions forming a Pareto front. This front consists exclusively of non-dominated solutions. A solution is considered non-dominated if no other solution exists that is superior in one objective without being inferior in the other. In other words, to improve a non-dominated solution with respect to one objective, a trade-off in the form of a degradation in the other objective must be accepted. Conversely, a dominated solution is one for which at least one other solution exists that offers better performance in one objective while being no worse in the other, making it an objectively suboptimal choice.

#### 3.6.3. Iterative Model Refinement and Candidate Selection

The framework employs an iterative backward pruning methodology to integrate feature selection directly into the optimization process. This automatic procedure relies, therefore, on both Dual-Objective Optimization and Iterative feature pruning (DOO-IT). The procedure begins with the complete descriptor set. A full DOO is executed, producing a Pareto front of non-dominated models. From this front, a single candidate model for the current iteration is selected, governed by the one standard error (1-SE) rule [102,103]. This involves identifying the most accurate model on the front and defining a performance threshold based on its standard error; the simplest model (lowest SV ratio) within this threshold is then chosen. Once a candidate is selected, its features are ranked based on permutation importance with 10 repeats [104]. The least impactful descriptor is then eliminated, and the procedure repeats with a new, full DOO on the reduced feature set. This cycle continues until a specified minimum number of features is reached, generating a series of robust, parsimonious candidate models at each level of complexity. The independent runs are performed 50 times for exploring the hyperparameters’ hyperspace for variety of train–test splits.

#### 3.6.4. Final Model Selection Using a Multi-Criteria Selection Scheme

In our previous paper [105], the final model selection was performed by plotting the corrected Akaike Information Criterion (AICc) [105,106,107,108] against the number of descriptors. However, the AICc is not well established for nuSVR regressors and introduces ambiguity. Hence, the theoretical criterion was replaced in this paper with a rigorous data-driven multi-criteria framework to select solutions that balance fit and parsimony. Hence, to objectively select the single best model from the family of candidates generated by the iterative procedure, a rigorous multi-criteria framework was designed to ensure robust predictive performance and chemical interpretability. After completing all the independent runs of the DOO-IT procedure, which generated numerous candidate models across varying descriptor complexities through 40 independent 80/20 training–test splits, a three-tiered selection strategy was applied. First, architectural optimization identified the optimal descriptor count through stability analysis, selecting models that consistently appeared across multiple runs while maintaining performance within one standard error of the minimum test MAE. This approach was inspired by the one standard error rule but it was enhanced with empirical stability thresholds (≥30% frequency per descriptor count), ensuring parsimonious model selection without relying on theoretically problematic information criteria. For the final model deployment, a composite scoring system was used that balanced predictive accuracy (50% weight), explanatory power (30% weight via R^2^), and generalization capability (20% weight via train–test performance gaps). From the architecturally optimal descriptor count, the specific model instance was selected that maximized the values of composite scores while demonstrating high descriptor stability—prioritizing molecular features consistently appearing across independent runs. This dual emphasis on both model architecture and specific feature set ensured that the deployed model not only preserved predictive performance but also embodied chemically meaningful and reproducible descriptor combinations. The final model was validated through comprehensive residual analysis, applicability domain assessment, and external validation where available, providing a transparent, empirically grounded foundation for practical solubility prediction in pharmaceutical and chemical development applications.

The DOO-IT framework was implemented as a fully automated pipeline using Python 3.10 [109] with the scikit-learn [110], Optuna [101], and pandas [111] libraries. To rigorously assess solution stability, the entire procedure was repeated fifteen independent times. Each dual-objective optimization within this process was configured to run for 2000 trials, ensuring a comprehensive exploration of the solution space.

## 4. Conclusions

This study addresses the challenge of solubility prediction, a problem of central importance in pharmaceutical and green chemistry research. Accurate predictive models therefore provide a powerful tool to reduce experimental workload, accelerate drug development pipelines, and enable the rational design of novel solvent systems such as DESs that combine efficiency with environmental compatibility. The Dual-Objective Optimization with Iterative feature pruning (DOO-IT) framework was applied for this task.

This study demonstrates that stability analysis of the DOO-IT framework uncovers not a single global optimum but two distinct regions of predictive excellence for modeling the solubility of pharmaceutical acids in deep eutectic solvents. On one side of the solution landscape lies an ultra-parsimonious eight-descriptor model that combines high predictive performance with minimal computational cost. This model integrates COSMO-RS logarithmic solubility as an anchor descriptor, which enables it to correct systematic deviations at solubility extremes and deliver near-perfect agreement with experimental values. By revealing two complementary “basins of excellence,” our analysis highlights the versatility of the DOO-IT framework in identifying multiple scientifically meaningful optima that balance accuracy, parsimony, and interpretability. The findings also extend our previous works, where a single model was sufficient to describe a narrower chemical space. In the present, more diverse dataset, the appearance of dual optimal regimes underscores the importance of tailoring model complexity to the scope of the prediction task. Taken together, these results suggest a pragmatic two-tiered strategy for future studies of solubility in deep eutectic solvents and related systems. Initial high-throughput screening can be effectively performed with the COSMO-RS-free parsimonious model, while subsequent high-precision evaluation of promising candidates can benefit from the expanded descriptor set that incorporates COSMO-RS calculations. This workflow balances efficiency with accuracy, making it possible to explore broader chemical spaces without sacrificing predictive reliability. Looking forward, validating the transferability of both models to other classes of solvents, as well as developing ensemble or adaptive strategies that dynamically combine parsimonious and high-performance regimes, will further enhance the applicability of this approach in green chemistry and pharmaceutical design.

## Data Availability

The original contributions presented in this study are included in the article/Supplementary Materials. Further inquiries can be directed to the corresponding author.

## Supplementary Materials

The following supporting information can be downloaded at https://www.mdpi.com/article/10.3390/molecules30224361/s1. Table S1. Mole-fraction solubility of mefenamic acid in choline chloride- or menthol-containing deep eutectic solvents (DESs). Table S2. Mole-fraction solubility of niflumic acid in choline chloride- or menthol-containing deep eutectic solvents (DESs). Table S3. List of descriptors used for models development. Table S4. List of references of solubility data [76,82,83,90].
