# Supplementary material for: Duality of Simplicity and Accuracy in QSPR: A Machine Learning Framework for Predicting Solubility of Selected Pharmaceutical Acids in Deep Eutectic Solvents
Source: Molecules. 2025 Nov 11;30(22):4361. doi: 10.3390/molecules30224361 (PMC12655841; doi:10.3390/molecules30224361)
Supplement: Supplementary file 1 [file molecules-30-04361-s001.zip › molecules-3932424-supplementary.pdf]

Supplementary Materials

Duality of Simplicity and Accuracy in QSPR: A Machine Learning Framework for Predicting Solubility of Selected Pharmaceutical Acids in Deep Eutectic Solvents

Piotr Cysewski *, Tomasz Jeliński, Julia Giniewicz, Anna Kaźmierska and Maciej Przybyłek

Department of Physical Chemistry, Faculty of Pharmacy, Collegium Medicum in Bydgoszcz, Nicolaus Copernicus University in Toruń, Kurpińskiego 5, 85-950 Bydgoszcz, Poland

Table of Contents

SI. Experimental solubility values obtained for mefenamic (MEF) and niflumic acids (NIF). 1

Tables S1 and S2. Mole-fraction solubility of mefenamic acid in choline chloride– or menthol–containing deep eutectic solvents (DESs). 1

Tables S3 and S4. Mole-fraction solubility of niflumic acid in choline chloride– or menthol–containing deep eutectic solvents (DESs). 2

S1. Experimental solubility values obtained for mefenamic (MEF) and niflumic acids (NIF).

Table S1. Mole-fraction solubility of mefenamic acid in choline chloride- or menthol-containing deep eutectic solvents (DESs).

Solvent	$x_{MEF} \times 10^4$				
	3:1	2:1	1:1	1:2	1:3
ChCl/P2D	-	-	3.88±0.12	8.20±0.36	4.16±0.21
ChCl/DEG	-	-	64.85±1.77	54.84±2.63	33.12±0.99
ChCl/TEG	-	-	2.99±0.11	5.32±0.21	5.26±0.21
ChCl/B3D	-	-	1.77±0.09	4.68±0.17	2.60±0.09
ChCl/GLY	-	-	2.42±0.11	45.76±2.18	2.35±0.09
ChCl/ETG	-	-	1.38±0.06	1.87±0.08	1.53±0.05
ChCl/TRG	-	-	4.10±0.16	3.44±0.14	3.13±0.11
Men/P2D	52.41±2.60	56.46±1.79	63.39±3.14	32.15±1.41	25.15±1.03
Men/DEG	56.75±3.01	56.04±2.52	62.52±2.39	57.33±1.86	48.01±2.16
Men/TEG	74.54±2.37	83.44±4.22	107.87±3.09	99.76±4.14	91.55±4.44
Men/B3D	42.89±1.30	47.82±1.79	40.25±2.05	27.60±1.15	23.09±1.09
Men/GLY	44.97±2.17	45.39±2.38	43.69±1.78	46.96±2.43	62.03±3.11
Men/ETG	48.51±2.30	39.63±1.30	32.52±1.64	28.95±1.16	31.15±1.69
Men/TRG	29.53±1.53	61.74±3.34	122.60±6.63	134.45±5.94	139.78±4.30

Table S2. Mole-fraction solubility of niflumic acid in choline chloride- or menthol-containing deep eutectic solvents (DESs).

Solvent	$x_{NIF} \times 10^4$				
	3:1	2:1	1:1	1:2	1:3
ChCl/P2D	-	-	8.29±0.24	2.38±0.10	4.11±0.17
ChCl/DEG	-	-	15.68±0.85	2.94±0.16	2.82±0.09
ChCl/TEG	-	-	9.14±0.29	8.39±0.31	5.77±0.27
ChCl/B3D	-	-	13.87±0.7	38.54±1.13	2.69±0.11
ChCl/GLY	-	-	3.89±0.16	11.01±0.39	3.02±0.08
ChCl/ETG	-	-	4.16±0.14	3.13±0.09	3.3±0.10
ChCl/TRG	-	-	146.72±7.27	23.22±1.26	9.37±0.50
Men/P2D	44.97±1.85	49.08±2.65	73.99±2.28	62.42±1.82	50.37±1.66
Men/DEG	16.92±0.76	53.86±2.21	91.03±4.79	107.38±3.89	100.68±3.37
Men/TEG	35.92±1.01	44.98±1.87	110.41±5.58	127.17±4.47	122.4±5.94
Men/B3D	23.8±0.92	26.96±1.35	73.13±1.94	63.11±1.71	57.22±2.24
Men/GLY	16.16±0.87	33.7±1.52	210.51±8.94	147.52±6.64	122.42±6.04
Men/ETG	33.67±1.13	39.03±0.84	73.06±2.51	50.75±1.96	33.61±1.10
Men/TRG	52.77±2.44	73.92±3.11	206.12±7.87	181.64±9.69	178.68±7.46

S2. Dataset and descriptors

Table S3. List of descriptors used for models development.

columns name	notation in text
$\log(x_{API}^{exp})$	experimental solubility
$\log(x_{API}^{COSMO})$	COSMOtherm computed solubility
dmu	$\Delta\mu$
dE_tot	ΔE_{int}
dE_Misfit	ΔE_{misfit}
dE_HB	ΔE_{HB}
dE_vdW	ΔE_{vdW}
mu1_sat	μ_{API}
E1_tot_sat	$E_{int,API}$
E1_Misfit_sat	$E_{misfit,API}$
E1_HB_sat	$E_{HB,API}$
E1_vdW_sat	$E_{vdW,API}$
mu_solvent	μ_{DES}
E_tot_solvent	$E_{int,DES}$
E_Misfit_solvent	$E_{misfit,DES}$
E_HB_solvent	$E_{HB,DES}$
E_vdW_solvent	$E_{vdW,DES}$
d-HBD1(12)	ΔHBD1 (from -0.03e/Å^2 to -0.025 e/Å^2)
d-HBD2(12)	ΔHBD2 (from -0.025e/Å2 to -0.020 e/Å2)
d-HBD3(12)	ΔHBD3 (from -0.020e/Å2 to -0.015 e/Å2)
d-HBD4(12)	ΔHBD4 (from -0.015e/Å2 to -0.010 e/Å2)
d-HH1(12)	ΔHH1 (from -0.010e/Å2 to -0.005 e/Å2)
d-HH2(12)	ΔHH2 (from -0.005e/Å2 to 0.000 e/Å2)
d-HH3(12)	ΔHH3 (from 0.000e/Å2 to +0.005 e/Å2)
d-HH4(12)	ΔHH4 (from +0.005e/Å2 to +0.010 e/Å2)
d-HBA1(12)	ΔHBA1 (from +0.010e/Å2 to +0.015 e/Å2)
d-HBA2(12)	ΔHBA2 (from +0.015e/Å2 to +0.020 e/Å2)
d-HBA3(12)	ΔHBA3 (from +0.020e/Å2 to +0.025 e/Å2)
d-HBA4(12)	ΔHBA4 (from +0.025e/Å2 to +0.030 e/Å2)

API-HBD1(12)	HBD1$_{API}$ (from -0.03e/Å^2 to -0.025 e/Å^2)
API-HBD2(12)	HBD2$_{API}$ (from -0.025e/Å^2 to -0.020 e/Å^2)
API-HBD3(12)	HBD3$_{API}$ (from -0.020e/Å^2 to -0.015 e/Å^2)
API-HBD4(12)	HBD4$_{API}$ (from -0.015e/Å^2 to -0.010 e/Å^2)
API-HH1(12)	HH1$_{API}$ (from -0.010e/Å^2 to -0.005 e/Å^2)
API-HH2(12)	HH2$_{API}$ (from -0.005e/Å^2 to 0.000 e/Å^2)
API-HH3(12)	HH3$_{API}$ (from 0.000e/Å^2 to +0.005 e/Å^2)
API-HH4(12)	HH4$_{API}$ (from +0.005e/Å^2 to +0.010 e/Å^2)
API-HBA1(12)	HBA1$_{API}$ (from +0.010e/Å^2 to +0.015 e/Å^2)
API-HBA2(12)	HBA2$_{API}$ (from +0.015e/Å^2 to +0.020 e/Å^2)
API-HBA3(12)	HBA3$_{API}$ (from +0.020e/Å^2 to +0.025 e/Å^2)
API-HBA4(12)	HBA4$_{API}$ (from +0.025e/Å^2 to +0.030 e/Å^2)
DES-HBD1(12)	HBD1$_{DES}$ (from -0.03e/Å2 to -0.025 e/Å2)
DES-HBD2(12)	HBD2$_{DES}$ (from -0.025e/Å2 to -0.020 e/Å2)
DES-HBD3(12)	HBD3$_{DES}$ (from -0.020e/Å2 to -0.015 e/Å2)
DES-HBD4(12)	HBD4$_{DES}$ (from -0.015e/Å2 to -0.010 e/Å2)
DES-HH1(12)	HH1$_{DES}$ (from -0.010e/Å2 to -0.005 e/Å2)
DES-HH2(12)	HH2$_{DES}$ (from -0.005e/Å2 to 0.000 e/Å2)
DES-HH3(12)	HH3$_{DES}$ (from 0.000e/Å2 to +0.005 e/Å2)
DES-HH4(12)	HH4$_{DES}$ (from +0.005e/Å2 to +0.010 e/Å2)
DES-HBA1(12)	HBA1$_{DES}$ (from +0.010e/Å2 to +0.015 e/Å2)
DES-HBA2(12)	HBA2$_{DES}$ (from +0.015e/Å2 to +0.020 e/Å2)
DES-HBA3(12)	HBA3$_{DES}$ (from +0.020e/Å2 to +0.025 e/Å2)
DES-HBA4(12)	HBA4$_{DES}$ (from +0.025e/Å2 to +0.030 e/Å2)

Table S4. List of references of solubility data.

solute	DOI
nefenamic acid	this work
niflumic acid	this work
ibuprofen	10.3390/molecules29102296
ketoprofen	10.3390/molecules29102296
ferulic acid	10.3390/molecules29163841
probenecid	10.1016/j.ijpharm.2019.118682
caffeic acid	submitted to IJMS
p-coumaric acid	submitted to IJMS
syryngic acid	submitted to IJMS
flufenamic acid	10.3390/molecules30163434
